# High-efficiency polymer solar cells with small photon energy loss

**DOI:** 10.1038/ncomms10085

**Published:** 2015-12-02

**Authors:** Kazuaki Kawashima, Yasunari Tamai, Hideo Ohkita, Itaru Osaka, Kazuo Takimiya

**Affiliations:** 1 Department of Applied Chemistry, Graduate School of Engineering, Hiroshima University, 1-4-1 Kagamiyama, Higashi-Hiroshima, Hiroshima 739-8527, Japan; 2 RIKEN Center for Emergent Matter Science (CEMS), 2-1 Hirosawa, Wako, Saitama 351-0198, Japan; 3 Department of Polymer Chemistry, Graduate School of Engineering, Kyoto University, Katsura, Kyoto 615-8510, Japan; 4 Precursory Research for Embryonic Science and Technology (PRESTO), Japan Science and Technology Agency, Tokyo 102-0075, Japan

## Abstract

A crucial issue facing polymer-based solar cells is how to manage the energetics of the polymer/fullerene blends to maximize short-circuit current density and open-circuit voltage at the same time and thus the power conversion efficiency. Here we demonstrate that the use of a naphthobisoxadiazole-based polymer with a narrow bandgap of 1.52 eV leads to high open-circuit voltages of approximately 1 V and high-power conversion efficiencies of ∼9% in solar cells, resulting in photon energy loss as small as ∼0.5 eV, which is much smaller than that of typical polymer systems (0.7–1.0 eV). This is ascribed to the high external quantum efficiency for the systems with a very small energy offset for charge separation. These unconventional features of the present polymer system will inspire the field of polymer-based solar cells towards further improvement of power conversion efficiencies with both high short-circuit current density and open-circuit voltage.

## Abstract (web summary)

Maximising short-circuit current density and open circuit voltage in polymer-fullerene solar cells is a critical issue. Here, the authors use an aphthobisoxadiazole-based polymer and observe a low photon energy loss of 0.5 eV, with an open-circuit voltage of 1 V and power conversion efficiency of 9%.

## Graphical abstract

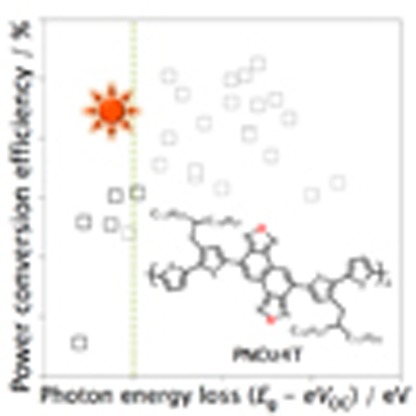

Polymer-based bulk-heterojunction solar cells (PSCs) that use semiconducting polymers and fullerene derivatives as p-type (hole-transport or electron donor) and n-type (electron-transport or electron acceptor) materials, respectively, are an emerging renewable energy technology that enables low-cost and low-environmental impact production and yields light-weight and flexible devices^1^,^2^,^3^. Drastic improvements of the power conversion efficiency (PCE) made in the last decade^4^,^5^,^6^,^7^,^8^,^9^ have resulted in PCEs surpassing 10% even in single-junction cells^10^,^11^. This has been achieved mainly by the development of new semiconducting polymers having donor–acceptor (D–A) backbones where electron-rich (donor; D) and electron-deficient (acceptor; A) units are combined, owing to their narrow optical bandgap^12^,^13^. Such polymers also offer great potentials in p-type polymer/n-type polymer (all-polymer) solar cells, which are recently resulted in PCEs more than 5% (refs 14, 15, 16).

To improve PCEs further, it is necessary to realize a high short-circuit current (*J*_SC_) and a high open-circuit voltage (*V*_OC_) at the same time. However, it is a well-known fact that there is a trade-off between those two parameters and thus, minimizing the trade-off is one of the most crucial issues. A key to resolving the issue in PSCs is to reduce the relatively large photon energy loss (*E*_loss_), which is defined by *E*_g_−*eV*_OC_, where *E*_g_ is the optical bandgap of the semiconducting polymer evaluated from the absorption edge^17^,^18^,^19^,^20^,^21^. The *E*_loss_ in recently reported high-efficiency PSCs is typically 0.7–1.0 eV (ref. 18), which is larger than those in inorganic solar cells and perovskite solar cells, which are around 0.4–0.5 eV (refs 19, 20). Very recently, some particular polymers were reported to have small *E*_loss_ in PSCs when used in combination with [6,6]-phenyl-C_61_-butyric acid methyl ester (PC_61_BM) or [6,6]-phenyl-C_71_-butyric acid methyl ester (PC_71_BM). Bazan and co-workers demonstrated that the use of a pyridylthiadiazole-based polymer reduced *E*_loss_ in the PSC to *ca.* 0.6 eV, which leads to a relatively high *V*_OC_ of 0.86 V even though the polymer has a narrow *E*_g_ of 1.47 eV (ref. 18). Shortly thereafter Janssen and co-workers showed that the use of thiazole-linked diketopyrrolopyrrole (DPP-2Tz) polymers, having a *E*_g_ of around 1.44–1.53 eV, reduced the *E*_loss_ below 0.55 eV, resulting in a high *V*_OC_ greater than 0.92 V (ref. 21). Although PSCs based on those polymers could potentially afford both high *J*_SC_ and *V*_OC_, the *J*_SC_ was in fact relatively low and PCEs were limited to 5–6% because of the trade-off.

The trade-off between *J*_SC_ and *V*_OC_ is also strongly related to the match of the molecular orbital energy levels between the polymer and fullerene. Given the fundamental working mechanism of PSCs, the realization of both high *J*_SC_ and *V*_OC_ requires that the polymer must have both a narrower *E*_g_ and a deeper highest occupied molecular orbital (HOMO) energy level^22^. However, this inevitably results in a deeper lowest unoccupied molecular orbital (LUMO) energy level, diminishing the energy offset of the LUMOs between the polymer and fullerene (Δ*E*_L_), which is considered as a driving force for the photoinduced charge separation. This eventually causes a loss of the driving force, in turn being detrimental to the PCE improvement. In this regard, synthetic chemists face a formidable challenge: to create new semiconducting polymers to manage the energetics between polymers and fullerenes and thus to minimize the trade-off.

Recently, we have reported^23^,^24^ that a D–A narrow bandgap polymer (PNTz4T, *E*_g_=1.56 eV) based on quaterthiophene and naphtho[1,2-c:5,6-c′]bis[1,2,5]thiadiazole (NTz) as the D and A units, respectively, showed as high as 10.1% PCE with a high *J*_SC_ of 19.4 mA cm^−2^ (ref. 25). On the other hand, *V*_OC_ was limited to 0.71–0.74 V because of the moderately deep HOMO energy level of –5.14 eV. As a result, *E*_loss_ in this system was 0.82–0.85 eV, which is typical of the semiconducting polymers. Naphtho[1,2-c:5,6-c′]bis[1,2,5]oxadiazole (NOz, ref. 24), which is a new fused heterocycle as an oxygen analogue of NTz and was also independently developed by Zhang *et al*.^26^, can deepen both the HOMO and LUMO energy levels compared with NTz while maintaining the *E*_g_, most likely as a result of the stronger electron negativity of oxygen than sulfur.

Here we highlight that the use of a D–A copolymer of quaterthiophene and NOz (PNOz4T, Fig. 1a)^24^, with a narrow *E*_g_ (1.52 eV) similar to that of PNTz4T and deeper HOMO and LUMO energy levels, affords high PCEs of up to 8.9% along with a remarkably high *V*_OC_ of ∼1 V in inverted PSCs combined with PCBM (PC_61_BM or PC_71_BM). Most importantly, the *E*_loss_ in this system is 0.52–0.56 eV, which is among the smallest values for PSCs and approaches the value for inorganic solar cells. We also note that, interestingly, the PNOz4T/PCBM system achieves such high PCEs despite the fact that Δ*E*_L_ is ∼0.1 eV, which is smaller than the empirical threshold of 0.3 eV. Furthermore, we find that through the spectroscopic studies, the small Δ*E*_L_ will not be a limiting factor in the charge separation process, and there may be still room for improving the PCE in this system. These results open doors for the solution of the trade-off issue and for the realization of PCEs exceeding 15%.

## Results

### Properties and energetics of the materials and the blend

**Figure 1:**
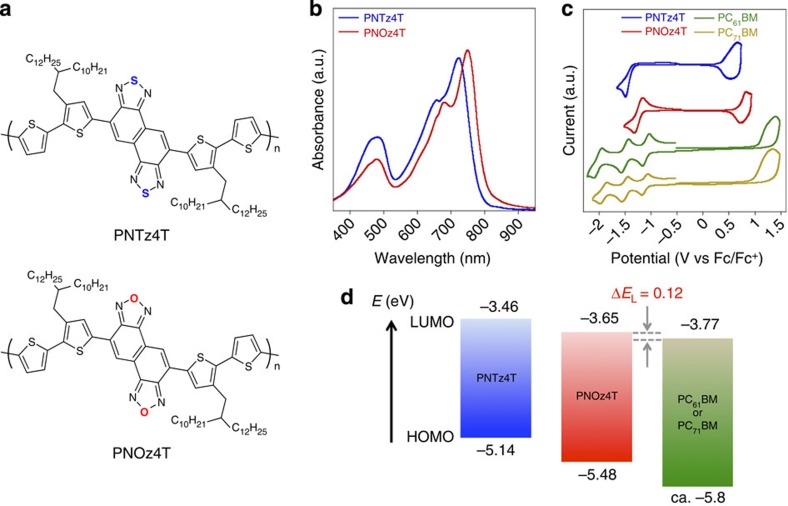
Structures and properties of the polymers. (**a**) Chemical structures of PNTz4T and PNOz4T. (**b**) Ultraviolet–visible absorption spectra of PNTz4T and PNOz4T in the thin film spin-coated from chlorobenzene solution. (**c**) Cyclic voltammograms of PNTz4T, PNOz4T (in the thin film), PC_61_BM and PC_71_BM (in the solution). (**d**) Energy diagrams of PNTz4T, PNOz4T, PC_61_BM and PC_71_BM, where the values were estimated by cyclic voltammetry. a.u., arbitrary unit.

PNOz4T was synthesized by polymerizing dibrominated dithienyl-NOz with long branched alkyl groups (2-decyltetradecyl) and distannylated bithiophene via the Stille coupling reaction (Supplementary Fig. 1 and Note 1)^24^. PNOz4T was soluble in hot chlorinated benzenes, such as chlorobenzene and *o*-dichlorobenzene. The number-average (*M*_n_) and weight-average (*M*_w_) molecular weights evaluated by gel permeation chromatography at 140 °C were 57.2 and 417 kDa, respectively, and the polydispersity index (PDI) was 7.3 (Supplementary Fig. 2). The large PDI is likely due to the strong aggregation nature of the polymer. The absorption spectra of PNTz4T and PNOz4T in the thin film are shown in Fig. 1b. PNOz4T provided a slightly red-shifted spectrum compared with PNTz4T. The absorption maximum (*λ*_max_) was observed at 749 nm, and the absorption edge (*λ*_edge_) was determined to be 815 nm. Both were 20–30 nm longer than those of PNTz4T (*λ*_max_=721 nm, *λ*_edge_=793 nm). *E*_g_ of PNOz4T was calculated to be 1.52 eV from *λ*_edge_, and was slightly smaller than that of PNTz4T (1.56 eV).

Figure 1c depicts the cyclic voltammograms of PNTz4T, PNOz4T, PC_61_BM and PC_71_BM. The HOMO and LUMO energy levels (*E*_H_ and *E*_L_) were determined using the redox onset potential (vs Fc/Fc^+^) of cyclic voltammograms, which were measured for five times for each material. *E*_H_ and *E*_L_ of PNOz4T were estimated to be −5.48 (±0.007) eV and −3.65 (±0.014) eV, which were 0.34 eV and 0.19 eV deeper than those of PNTz4T (*E*_H_=–5.14 (±0.022) eV, *E*_L_=–3.46 (±0.022) eV). The results indicate the strong electron deficiency of PNOz4T, which is consistent with the computation (Supplementary Fig. 3). *E*_H_s of PC_61_BM and PC_71_BM were –5.85 (±0.019) eV and –5.77 (±0.016) eV, respectively. *E*_L_ of –3.77 eV was obtained for both PC_61_BM and PC_71_BM (s.d. were ±0.015 and ±0.024, respectively). The values for PCBMs agree well with the reported values^27^. Figure 1d displays the energy diagrams of PNTz4T, PNOz4T and PCBM blend system using *E*_H_s and *E*_L_s. It is noted that whereas Δ*E*_L_ for PNTz4T (between PCBMs) is 0.31 eV, that for PNOz4T is 0.12 eV, which is much smaller than the widely referenced empirical threshold value of 0.3 eV (ref. 28). This indicates that the driving force of the photoinduced charge separation is reduced in the PNOz4T system compared with the PNTz4T system. Note that, however, the estimation of the energetics based on *E*_H_ and *E*_L_ evaluated by the measurements on the isolated polymer and PCBMs is considered to neglect the influence of exciton binding energy and other interfacial effects^29^,^30^. Alternatively, the energy offset can be estimated by the difference of the excited state energy of the polymer and the charge transfer (CT) state energy (*E*_CT_) of the polymer/PCBM blend^17^,^31^. Thus, we attempted to determine *E*_CT_ of the blend films by means of the electroluminescence measurement. However, for the PNOz4T/PC_71_BM system, the emission band of the pure PNOz4T and the CT emission band of the PNOz4T/PC_71_BM blend system measured by the electroluminescence cannot be distinguished (Supplementary Fig. 4), and thus *E*_CT_ cannot be evaluated. This suggests that the excited state of the polymer and the CT state of the blend are energetically very close, and thereby the energy offset is very small^32^. This is consistent with the small Δ*E*_L_ for the PNOz4T/PC_71_BM blend system measured by cyclic voltammetry. This will be further examined later in this paper.

### Solar cell properties

**Figure 2:**
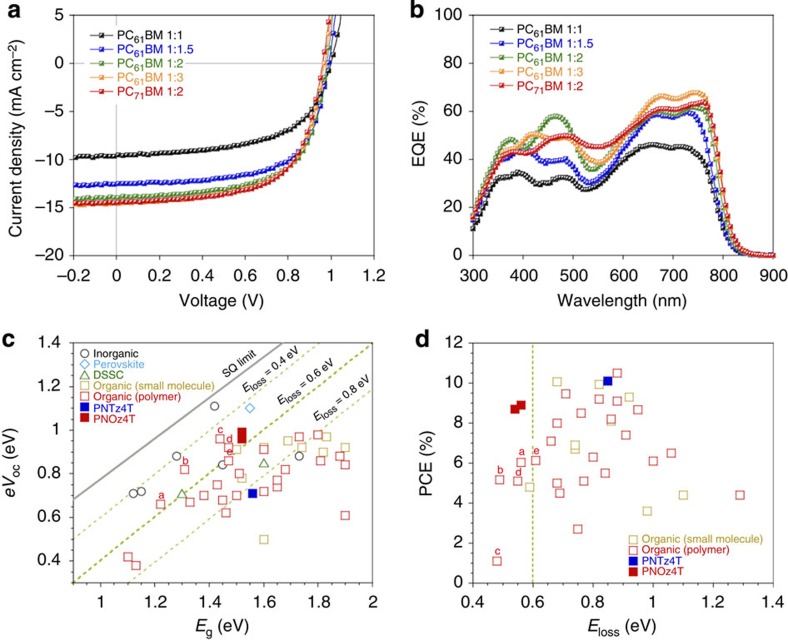
Photovoltaic performance of the PNOz4T-based cells. (**a**) *J*–*V* curves of PNOz4T/PC_61_BM cells with different polymer to PC_61_BM weight (p/n) ratios and a PNOz4T/PC_71_BM cell with a p/n ratio of 1:2. (**b**) EQE spectra of PNOz4T/PC_61_BM cells with different polymer to PC_61_BM weight (p/n) ratios and a PNOz4T/PC_71_BM cell with a p/n ratio of 1:2. (**c**) Plots of *eV*_OC_ against *E*_g_, which is determined from the absorption onset, for various solar cell systems. The grey line and green dotted line show *E*_loss_ calculated from the Shockley–Queisser (SQ) theory and *E*_loss_=0.4, 0.6, and 0.8 eV. (**d**) Plots of PCE against *E*_loss_ (=*E*_g_−*eV*_OC_) for organic solar cells (small molecules and polymers (PSCs)) extracted from **c**. (see Supplementary Table 2 for the detail of **c** and **d**) The red open squares **a**–**e** indicate the polymer systems with *E*_loss_≤0.6 eV.

The solar cells were fabricated using an inverted architecture, indium–tin-oxide (ITO)/ZnO/PNOz4T:PCBM/MoO_*x*_/Ag, where the active layer was spin-coated from the blend solution in chlorobenzene, and the optimal active layer thickness was around 200 nm. Figure 2a,b depict the current density (*J*)–voltage (*V*) curves and the external quantum efficiency (EQE) spectra of the cells, respectively, and Table 1 summarizes the photovoltaic parameters. Although the driving force of photoinduced charge separation, Δ*E*_L_, is smaller than the empirical threshold, the PNOz4T cells exhibited fairly high PCEs. In the cells with the PNOz4T to PC_61_BM weight (p/n) ratio of 1:1, a significantly high *V*_OC_ of 1.0 V was obtained (*J*_SC_=9.6 mA cm^–2^, FF=0.59, PCE=5.6%). It should be mentioned that the *V*_OC_ of 1.0 V is difficult to be achieved even with middle-to-wide bandgap polymers (Fig. 2c). With the increase of the PC_61_BM ratio to 1:1.5–1:3, although *V*_OC_ was slightly decreased, a marked increase in *J*_SC_ was observed and PCE was as high as 8.8% (*J*_SC_=14.6 mA cm^–2^, *V*_OC_=0.97 V, FF=0.62) at the p/n ratio of 1:3. The difference in PCE between the p/n ratios of 1:2 and 1:3 was marginal. The maximum PCE in the PNOz4T cell was 8.9% (*J*_SC_=14.5 mA cm^–2^, *V*_OC_=0.96 V, FF=0.64), which was obtained when PC_71_BM was used at the p/n ratio of 1:2. *J*–*V* curves of the PNOz4T/PC_71_BM cells and the corresponding photovolataic parameters are summarized in Supplementary Fig. 5 and Table 1, respectively. The observed *V*_OC_s of 0.96–1.0 V are higher by more than 0.2 V than those observed for the PNTz4T cells (*V*_OC_=0.71–0.74 V) (ref. 25), consistent with the difference in the HOMO energy level.

Importantly, as *E*_g_ of PNOz4T was 1.52 eV and *V*_OC_s of the PNOz4T-based cells were 0.96–1.0 V, *E*_loss_s of the PNOz4T/PCBM system were found to be 0.52–0.56 eV, which are significantly reduced from that of the PNTz4T/PCBM system (0.82–0.85 eV) (refs 23, 25) and are even smaller than 0.6 eV that has been referenced as the empirical limit for PSCs^17^. Figure 2c depicts the plots of *eV*_OC_ against *E*_g_ for the PNTz4T and PNOz4T systems in comparison with various solar cell systems such as inorganic, perovskite, dye-sensitized (DSSC), small molecule-based organic and PSC solar cells, in which the lines of *E*_loss_ estimated by the Shockley–Queisser theory^33^ and the empirically estimated limit (0.6 eV) are drawn. *E*_g_ for all the small molecular and polymer materials plotted here are defined as the value at the onset of film absorption (Supplementary Note 2). Figure 2d shows the plots of PCE against *E*_loss_ for the PNTz4T and PNOz4T systems along with other organic systems plotted in Fig. 2c. These plots were produced referring to the literatures by Bazan^18^ and Janssen^21^, and the corresponding data are summarized in Supplementary Table 2. The red squares of a–e indicate the polymer systems with *E*_loss_ ≤0.6 eV (refs 18, 21, 34, 35). It is clear that the *E*_loss_ of the PNOz4T system are among the lowest values reported so far for PSCs^18^,^21^, while the typical values for organic systems are 0.7–1.0 eV (Fig. 2c). It is noted that the *E*_loss_ of the PNOz4T system approach the values for inorganic or perovskite solar cells, 0.4–0.5 eV (refs 19, 20). More importantly, PCEs of the PNOz4T system are in fact the highest values among the organic systems having *E*_loss_ ≤0.6 eV (Fig. 2d). Such small *E*_loss_, as well as the fact that the high *V*_OC_ are achievable even with the narrow *E*_g_ of 1.52 eV, observed in PNOz4T cells are attributed to the very small Δ*E*_L_ of 0.12 eV.

**Figure 3:**
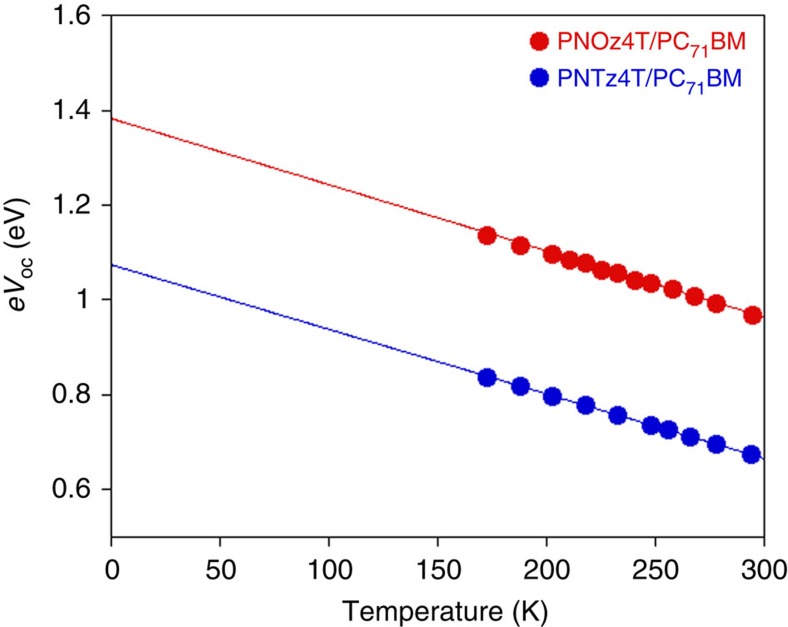
Temperature dependent *eV*_OC_ for a PNOz4T/PC_71_BM cell and a PNTz4T/PC_71_BM cell. The p/n ratio is 1:2 for both cells.

It has been known that the study of temperature-dependent *V*_OC_ allows us to evaluate the effective bandgap (*E*_g_^eff^) of the blend system that is a good measure of *E*_CT_ (refs 36, 37, 38). Thus, by evaluating *E*_g_^eff^, the energy offset between the polymer excited state and the CT state, can be estimated. We measured the *J–V* characteristics of a PNOz4T/PC_71_BM (1:2) cell in comparison with a PNTz4T/PC_71_BM (1:2) cell by varying the temperature from 294 to 173 K (Supplementary Fig. 6). Figure 3 shows the plots of *eV*_OC_ against temperature for both cells. Note that the *V*_OC_ values were corrected by considering the temperature dependence in *J*_SC_. The data were fitted by linear relation, and *E*_g_^eff^ was determined to be 1.38 and 1.07 eV for the PNOz4T and PNTz4T systems, respectively, from the intersection at 0 K. For the energy of the polymer-excited state, we use *E*_g_ following the previous studies by several groups^17^,^32^,^39^. Thus, the energy offset defined as *E*_g_−*E*_CT_ (*E*_g_^eff^) is 0.14 eV for the PNOz4T system and 0.49 eV for the PNTz4T system. Note that since *E*_g_^eff^ is determined at 0 K, *E*_CT_ at room temperature is typically larger by 0.1–0.2 eV than *E*_g_^eff^ (refs 36, 37) and thus the actual *E*_g_−*E*_CT_ is presumed to be negligibly small (∼0 eV) for the PNOz4T system. This is consistent with our observation that the electroluminescence of the PNOz4T/PC_71_BM blend system is almost the same as that of the PNOz4T neat film as mentioned before. By considering the small Δ*E*_L_=0.12 eV estimated by electrochemistry and the negligibly small *E*_g_−*E*_CT_ ∼0 eV, we can safely say that the driving force for the charge separation is quite small in the PNOz4T/PCBM system^32^,^39^.

The PNOz4T cells showed EQEs surpassing 60% at the polymer *λ*_max_ region, except for the cells that used PC_61_BM with the p/n ratio of 1:1. The highest EQE of 66% was obtained when PC_61_BM was used with a p/n ratio of 1:3. These EQE values for the PNOz4T cells were lower than those for the PNTz4T cells (EQE ∼75% at *λ*_max_), which reflects the lower *J*_SC_ despite the fact that PNOz4T has an absorption range, that is, *E*_g_, similar to that for PNTz4T. This issue will be further discussed later in this paper. We note here that, however, these EQE values are quite high for the polymer/PCBM systems with such a small energy offset, that is, Δ*E*_L_ or *E*_g_−*E*_CT_. For instance, although the system of DPP-2Tz polymers/PC_71_BM was reported to have a small Δ*E*_L_ of 0.09–0.21 eV and provide a high *V*_OC_ of more than 0.9 V, it only showed low EQEs of 5–40%, which resulted in a low *J*_SC_ of 2.0–8.8 mA cm^−2^ (ref. 21). Similarly high EQEs with the small energy offset were previously reported by Vandewal and co-workers, in which isoindigo-based polymers with *E*_g_−*E*_CT_ of ∼0.0 and 0.1 eV showed internal quantum efficiencies of ∼45% (EQE ∼30%) and 87% (EQE ∼60%) (ref. 32). However, in this case, PCEs were limited to 4.5% (*V*_OC_=0.91 V) (ref. 40) and 6.3% (*V*_OC_=0.70 V), respectively^41^. Therefore, to the best of our knowledge, in demonstrating high PCEs of ∼9% with a high *V*_OC_ of ∼1 V, small *E*_loss_ and a small energy offset at the same time, PNOz4T is regarded as quite a unique narrow bandgap polymer.

### Charge transport properties

The charge carrier mobility of PNOz4T was evaluated for hole-only devices (ITO/PEDOT:PSS/PNOz4T:PCBM/MoO_*x*_/Ag) by using the space-charge-limited current model, where the p/n ratios were 1:3 and 1:2 for PC_61_BM and PC_71_BM, respectively (Supplementary Fig. 7a). Blend films with PC_61_BM and PC_71_BM exhibited hole mobilities of 2.4 × 10^–3^ cm^2^ V^−1^ s^−1^ and 1.7 × 10^–3^ cm^2^ V^−1^ s^−1^, respectively, which are high for semiconducting polymers. To confirm the balance of the charge carrier transport between hole and electron, we also fabricated electron-only devices using the blend films (ITO/ZnO/PNOz4T:PCBM/LiF/Al) with the same p/n ratios as the hole-only devices (Supplementary Fig. 7b). Electron mobility was also of order 10^–3^ cm^2^ V^−1^ s^−1^; it was 2.6 × 10^–3^ and 1.5 × 10^–3^ cm^2^ V^−1^ s^−1^ for the blend films with PC_61_BM and PC_71_BM, respectively. These results indicate that the hole and electron transports are well-balanced in the PNOz4T/PCBM blend films.

### Thin film characterization

**Figure 4:**
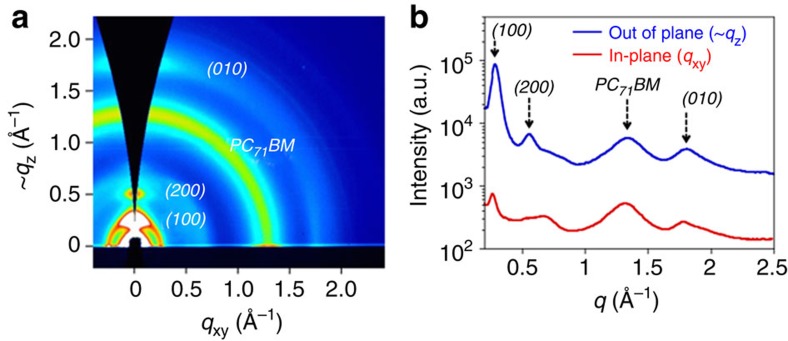
2D GIXD characterization of the PNOz4T/PC_71_BM blend film spun on the ZnO-coated glass/ITO substrate (1:2 wt ratio). (**a**) 2D GIXD image. (**b**) Cross-sectional profiles of the 2D GIXD image along the *q*_z_ (out of plane) and *q*_xy_ (in-plane) axes. a.u., arbitrary unit.

The ordering structure of PNOz4T in the thin film blended with PC_71_BM (p/n ratio=1:2) was investigated by grazing incidence wide-angle diffraction (GIXD) measurements (Fig. 4)^42^. The two-dimensional diffraction (2D GIXD) image provided diffractions corresponding to the lamellar structure, (*h*00), and the π–π stacking structure, (010), at the small-angle region and the wide-angle region, respectively, which are typical of semiconducting polymers (Fig. 4a)^43^. Both the lamellar and the π–π stacking diffractions are present along both the *q*_z_ and *q*_xy_ axes, indicative of the coexistence of edge-on and face-on orientations where the backbone plane stands and lies flat on the substrate, respectively. The π–π stacking distance was determined to be 3.5 Å (*q*∼1.8 Å^−1^) from the cross-sectional profiles of the 2D GIXD image (Fig. 4b), which is narrow for semiconducting polymers. The PNOz4T/PC_61_BM thin film displayed 2D GIXD image similar to that of the PNOz4T/PC_71_BM thin film (Supplementary Fig. 8). The GIXD study suggests that PNOz4T forms a highly crystalline structure in the blend film, which, together with the presence of face-on crystallites, is in good agreement with the high out-of-plane mobility and thus the high photovoltaic performance of PNOz4T.

**Figure 5:**
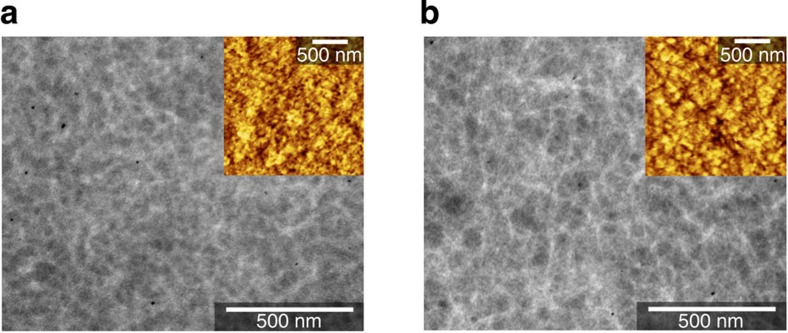
TEM images of the polymer/PC_71_BM blend film (1:2 wt ratio). (**a**) PNTz4T/PC_71_BM thin film. (**b**) PNOz4T/PC_71_BM thin film. Inset displays the AFM images of the corresponding films.

Transmission electron microscopy (TEM) and atomic force microscopy (AFM) images of PNTz4T/PC_71_BM and PNOz4T/PC_71_BM blend films with the p/n ratio of 1:2 are shown in Fig. 5. The TEM images revealed good phase separation with bicontinuous networks for both polymer systems^44^. It is noted that the PNOz4T/PC_71_BM film gave relatively large domains compared with the PNTz4T/PC_71_BM film. The domain sizes (fibril widths) of the PNTz4T/PC_71_BM and PNOz4T/PC_71_BM films were roughly 15–30 and 20–60 nm, respectively. The AFM images in the inset revealed a texture with well phase-separated domains. Similar to the TEM images, the PNOz4T/PC_71_BM film gave slightly larger domains than the PNTz4T/PC_71_BM film. This is probably because PNOz4T has stronger aggregation property than PNTz4T.

### Photoluminescence quenching study

**Figure 6:**
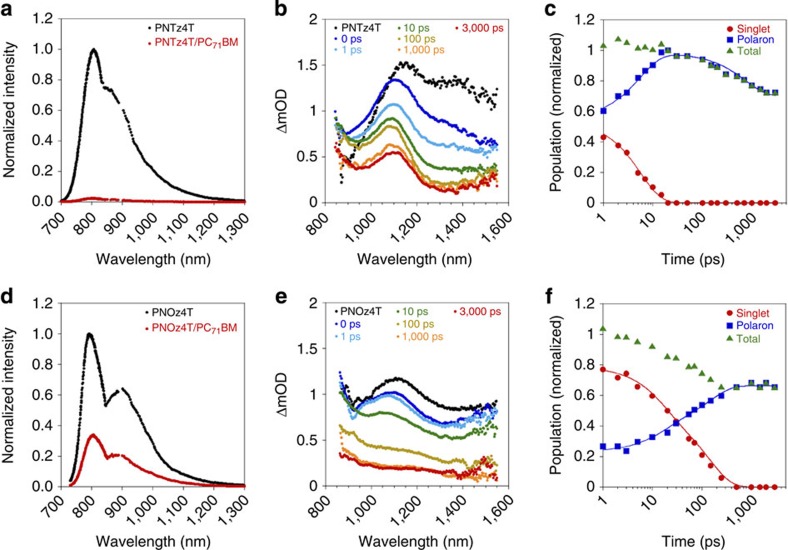
Spectroscopic studies of the polymer and blend films (1:2 wt ratio). (**a**,**d**) Normalized photoluminescence spectra; (**a**) PNTz4T neat film and PNTz4T/PC_71_BM blend film excited at 630 nm. (**d**) PNOz4T neat film and PNOz4T/PC_71_BM blend film excited at 700 nm. (**b**,**e**) Femtosecond transient absorption spectra; (**b**) PNTz4T neat film measured at 0 ps (black dots shown as PNTz4T) and the PNTz4T/PC_71_BM blend film measured at 0–3,000 ps. (**e**) PNOz4T neat film measured at 0 ps (black dots shown as PNOz4T) and the PNOz4T/PC_71_BM blend film measured at 0–3,000 ps. (**c**,**f**) Normalized femtosecond transient absorption signals; (**c**) PNTz4T/PC_71_BM blend film. (**f**) PNOz4T/PC_71_BM blend film. Polaron signals were extracted by subtracting the signals of singlet excitons.

To study the charge generation dynamics, we measured the photoluminescence spectra using the polymer neat film and the PC_71_BM blend film with the p/n ratio of 1:2 for PNTz4T and PNOz4T. Figure 6a,d depict the photoluminescence spectra of the PNTz4T and PNOz4T systems, respectively. Whereas photoluminescence of PNTz4T was almost completely quenched in the blend film (quenching efficiency >95%), photoluminescence of PNOz4T was still observed, where the photoluminescence quenching efficiency was as low as *ca.* 66±7%. This indicates that the excitons generated in PNOz4T are not completely dissociated into charge carriers.

### Transient absorption spectroscopy

We also measured the time evolution of the transient absorption spectra within the picoseconds time range^45^ using the polymer neat film and the PC_71_BM blend film with a p/n ratio of 1:2 for PNTz4T and PNOz4T. Figure 6b,e depict transient absorption spectra of the PNTz4T and PNOz4T blend films, respectively, together with those of the neat films (Supplementary Fig. 9). In transient absorption measurements under relatively strong excitation intensities, bimolecular deactivation processes such as singlet–singlet exciton annihilation and non-geminate charge recombination can produce additional (and not essential in this time range under the 1 Sun condition) decay channnels and lead to a faster decay. To avoid these processes, measurements were carried out under a weak excitation intensity whenever possible. Immediately after the laser excitation, a transient absorption band was observed at around 1,100–1,400 nm for both PNTz4T and PNOz4T (black dots), which is ascribed to polymer singlet excitons. This exciton band decayed in 1 ns. Instead, a long-lived absorption band was observed at around 1,100 nm, which is ascribed to polymer polarons. The detailed assignments are described in Supplementary Note 3 and Figs 9–13. As shown in Fig. 6c,f, the time evolution of each transient species can be extracted from the spectral simulation using those absorption spectra. The polaron signal was observed even at 0 ps for both the PNTz4T and PNOz4T films, indicative of the immediate charge generation on laser irradiation. In other words, exciton diffusion is negligible for such an immediate charge generation. This suggests that there exist regions in which the polymers and PC_71_BM are well mixed, and/or that polymer crystalline domains are sufficiently small for excitons to contact the p/n interface^46^. The large population of polarons at 0 ps in the PNTz4T film (*ca.* 0.6) compared with that in the PNOz4T film (*ca.* 0.2) suggests that the PNTz4T film has a larger fraction of mixed regions and/or small domains.

For both blend films, the singlet excitons decayed with the same time constant as the rise constant of polaron signals on a time scale of tens of picoseconds. This delayed charge generation is due to exciton diffusion in the relatively large cystalline domains of polymers. The time constant was 5.5 ps for the PNTz4T/PC_71_BM film, which is longer than that for typical high-performance D–A polymers^47^,^48^,^49^. Notably, the time constant was as long as 100 ps for the PNOz4T/PC_71_BM film, suggesting that the domain size of the polymer in the PNOz4T/PC_71_BM film is much larger than that in the PNTz4T/PC_71_BM film. This is consistent with the difference observed in the TEM images. Furthermore, this time constant is far longer than that reported for the regioregular poly(3-hexylthiophene) system, a familiar crystalline polymer, of around 10–30 ps^46^,^50^. Taking into account that the lifetime of the PNOz4T singlet exciton was 230 ps measured from the transient absorption decay of the neat film, the exciton quenching efficiency was estimated to be ∼57%, which roughly agrees with the quenching efficiency (∼66%) obtained in the photoluminescence measurement. These results indicate that ∼1/3 of the generated excitons are deactivated before arriving at the polymer/fullerene interface.

We note differences in charge recombination dynamics between PNTz4T and PNOz4T systems. For the PNTz4T/PC_71_BM film, the polaron signal decayed after 100 ps, and the decay was independent of the excitation intensity (Supplementary Fig. 11). We, therefore, ascribe the polaron decay to the geminate recombination^51^. On the other hand, no polaron decay was observed for the PNOz4T/PC_71_BM film up to a few nanoseconds, indicative of negligible geminate recombination. The geminate recombination in the PNTz4T system is partly due to the large fraction of the mixed region, which would enhance the recombination. The negligible recombination loss suggests that the PNOz4T system has potential advantage over the PNTz4T system.

## Discussion

We discuss the origin of the relatively low EQE (∼60%) and *J*_SC_ (below 15 mA cm^–2^) of the PNOz4T/PCBM system in comparison with those of the PNTz4T/PCBM system. The photoluminescence spectrum of the PNOz4T/PC_71_BM blend film showed that the photoluminescence signal for PNOz4T was not completely quenched. This suggests that the exciton diffusion to and/or the charge transfer at the polymer/fullerene interface are the main limiting process for the low EQE. This is consistent with the delayed charge generation with a time constant of as long as 100 ps observed in the transient absorption measurement. On the other hand, the TEM images clearly showed that the PNOz4T/PC_71_BM blend film exhibited larger phase-separated domains than the PNTz4T/PC_71_BM blend film. These findings suggest that the low efficiency of the PNOz4T system originates mainly from the exciton diffusion loss because of the large domain size. This is further supported by the observation of the polaron signals at 0 ps, which suggests that the energetical driving force for the charge separation is sufficient. We note here that in the PNOz4T/PC_71_BM films, transient absorption was measured at an excitation wavelength of 800 nm, which is around *λ*_edge_ of PNOz4T, to generate relaxed singlet excitons with no excess energy. In other words, charge generation via hot excitons can be ruled out. We, therefore, believe that the small Δ*E*_L_ and *E*_g_−*E*_CT_ in this system would not be a limiting factor but be rather sufficient for the photoinduced charge generation. This implies that further optimization of the phase separation structure can lead to the improvement of charge generation and thus overall PCE in this system.

In summary, we have shown that the use of PNOz4T, an NOz-based polymer, demonstrates as high as 8.9% PCE in single-junction inverted cells, along with a remarkably high *V*_OC_ of up to ∼1 V despite the fact that the polymer had a narrow bandgap of 1.52 eV. We primarily highlighted that the *E*_loss_ in this system, 0.52–0.56 eV, were found to be among the smallest values for PSCs, which were smaller than the empirical limit of 0.6 eV referenced to date, and were close to the values for inorganic solar cells. Another interesting feature is that, closely related to the small *E*_loss_, the energy offset (Δ*E*_L_ and *E*_g_−*E*_CT_) that is a driving force for the photoinduced charge separation is very small. In particular, Δ*E*_L_, a widely referenced important parameter in designing p-type semiconducting polymers, was 0.12 eV, which is much smaller than the empirical threshold value of 0.3 eV for efficient photoinduced charge separation. Overall, to the best of our knowledge, PNOz4T is the highest performing narrow bandgap polymer in terms of having several unconventional aspects at the same time: *V*_OC_ of ∼1 V and *E*_loss_ of ∼0.5 eV (and almost negligible energy offset), which are among the highest and smallest values for PSCs reported so far, respectively. Our study of charge generation dynamics in comparison with the PNTz4T system revealed that the relatively low EQE (∼66%) of the PNOz4T system originates in the relatively large domain size of the blend films and not in the small Δ*E*_L_. Thus, it is expected that further modification of the polymer structure and/or optimization of the morphology of the blend film can still improve EQE and thus *J*_SC_. Although further studies are necessary to understand why such a high performance is achievable even with a small energy offset, our results open the door to the realization of both high *J*_SC_ and *V*_OC_ at the same time, and thus the high PCE such as 15% in single-junction cells.

## Methods

### Materials

PC_61_BM and PC_71_BM were purchased from Frontier Carbon. PNTz4T (ref. 23) and PNOz4T (ref. 24) were synthesized according to the literature.

### Ultraviolet–visible absorption spectroscopy and cyclic voltammetry

Ultraviolet–visible absorption spectra were measured on a Shimadzu UV-3600 spectrometer. Cyclic voltammograms were recorded on an ALS electrochemical analyser Model 612D. Measurement of the polymers was carried out using films spun on the ITO glass in acetonitrile containing tetrabutylammonium hexafluorophosphate (Bu4NPF_6_, 0.1 M) as supporting electrolyte at a scan rate of 100 mV s^−1^. Measurement of PCBMs was carried out in *o*-dichlorobenzene/acetonitrile (4/1) solution. ITO and Pt were used as the working electrode for polymers and PCBMs, respectively. The counter electrode was made of Pt, and the reference electrode was Ag/AgCl. All the potentials were calibrated with the standard ferrocene/ferrocenium redox couple (Fc/Fc+: *E*_1/2_=+0.45 V measured under identical conditions).

### Solar cell fabrication and measurements

ITO substrates were pre-cleaned sequentially by sonicating in a detergent bath, de-ionized water, acetone and isopropanol at room temperature, and in a boiled isopropanol bath, each for 10 min. Then, the substrates were subjected to UV/ozone treatment at room temperature for 20 min. The pre-cleaned ITO substrates masked at the electrical contacts were coated with ZnO precursor by spin coating (3,000 r.p.m. for 30 s) a precursor solution prepared by dissolving zinc acetate dehydrate (0.5 g) and ethanolamine (0.14 ml) in 5 ml of 2-methoxyethanol. They were then baked in air at 200 °C for 30 min, rinsed with acetone and isopropanol, and dried in a glove box. The photoactive layers were deposited in a glove box (KOREA KIYON, KK-011AS-EXTRA) by spin coating: the chlorobenzene solution containing 4–5 g l^−1^ of the polymer sample with respective amount of PC_61_BM or PC_71_BM was kept at 140 °C for 30 min, and the hot solution was directly spin-coated on the substrate at 600 r.p.m. for 20 s. The thin films were transferred into a vacuum evaporator (ALS Technology, E-100 J) connected to the glove box. MoO_*x*_ (7.5 nm) and Ag (100 nm) were deposited sequentially by thermal evaporation under ∼10^–5^ Pa, where the active area of the cells was 0.16 cm^2^.

*J*–*V* characteristics of the cells were measured with a Keithley 2400 source measure unit in nitrogen atmosphere under 1 Sun (AM1.5G) conditions using a solar simulator (SAN-EI Electric, XES-40S1). The light intensity for the *J*–*V* measurements was calibrated with a reference PV cell (Konica Minolta AK-100 certified by the National Institute of Advanced Industrial Science and Technology, Japan). Temperature dependence of the *J*–*V* characteristics was measured with a direct-current voltage and current source/monitor (Advantest, R6243) in a vacuum prober system (ALS Technology, VPS3-50) under the illumination from a 100 W Xe lamp (Asahi Spectra, LAX-C100) equipped with a uniform illumination lens unit (Asahi Spectra, RLQL80-0.5). EQE spectra were measured with a spectral response measuring system (Soma Optics, Ltd., S-9241). The thickness of the active layer was measured with an AlphaStep D-100 surface profiler (KLA Tencor).

### Thin film characterization

GIXD experiments were conducted at the SPring-8 on beamline BL46XU. The samples were prepared in the same manner as actual devices on ZnO-coated ITO substrates. The samples were irradiated with an X-ray energy of 12.39 keV (*λ*=1 Å) at a fixed incidence angle on the order of 0.12° through a Huber diffractometer. The GIWAXD patterns were recorded with a 2D image detector (Pilatus 300 K). TEM measurements were performed using a JEOL JEM-1230 instrument. The blend films for the TEM measurement were prepared in the same manner as actual devices but on PEDOT:PSS-coated ITO substrates. Then, the substrates were transferred to TEM grids using the contact film-transfer method^52^,^53^. AFM measurements were carried out with actual devices using a Nanocute scanning probe microsope system (SII Nanotechnology, Inc.).

### Photoluminescence spectroscopy

Photoluminescence spectra were measured with a fluorescence spectrometer (Horiba Jobin Yvon, NanoLog) equipped with a photomultiplier tube (Hamamatsu, R928P) and a liquid-nitrogen-cooled InGaAs near-infrared array detector (Horiba Jobin Yvon, Symphony II) under ambient atmosphere. The excitation wavelength was set at 630 nm for PNTz4T/PC_71_BM and at 700 nm for PNOz4T/PC_71_BM.

### Transient absorption spectroscopy

Femtosecond transient absorption data were collected with a pump and probe femtosecond transient spectroscopy system. This system consists of a regenerative amplified Ti:sapphire laser (Spectra-Physics, Hurricane) and a transient absorption spectrometer (Ultrafast systems, Helios). The amplified Ti:sapphire laser provided 800 nm fundamental pulses at a repetition rate of 1 kHz with an energy of 0.8 mJ and a pulse width of 100 fs (full-width at half-maximum), which were split into two optical beams with a beam splitter to generate pump and probe pulses. One fundamental beam was converted into white-light pulses employed as probe pulses in the wavelength region from 400 to 1,700 nm. The other fundamental beam was used as pump pulses at 800 nm (for PNOz4T/PC_71_BM), at 760 nm (for PNTz4T/PC_71_BM) or at 750 nm (for neat films) after conversion with an ultrafast optical parametric amplifer (Spectra-Physics, TOPAS). The pump pulses were modulated mechanically at a repetition rate of 500 Hz. Temporal evolution of the probe intensity was recorded with a CMOS linear sensor (Ultrafast Systems, SPEC-VIS) for visible measurements, and with an InGaAs linear diode array sensor (Ultrafast Systems, SPEC-NIR) for near-infrared measurements. Transient absorption spectra and decays were collected over the time range of −0.5 ps to 3 ns. Typically, 2,500 laser shots were averaged at each delay time to obtain a detectable absorbance change as small as ∼10^−4^. To cancel out orientation effects on the dynamics, the polarization direction of the linearly polarized probe pulse was set at the magic angle of 54.7° with respect to that of the pump pulse. The sample films were sealed in a quartz cuvette purged with N_2_. Note that the transient absorption spectra and dynamics were highly reproducible even after several measurements. In other words, the laser irradiation had negligible effects on the sample degradation at least under those experimental conditions. Method for the microsecond transient absorption spectroscopy are described in Supplementary Methods.

## Additional information

**How to cite this article:** Kawashima, K. *et al*. High-efficiency polymer solar cells with small photon energy loss. *Nat. Commun.* 6:10085 doi: 10.1038/ncomms10085 (2015).

## Supplementary Material

Supplementary Information Supplementary Figures 1-13, Supplementary Tables 1-2, Supplementary Notes 1-3, Supplementary Methods and Supplementary References.

## Figures and Tables

**Table 1:** Photovoltaic parameters of PNOz4T-based solar cells.

**PCBM**	**p/n ratio**	Thickness (nm)*	***J***_**SC**_ **(mA cm**^−2^**)**†	***J***_**SC**_^EQE^ **(mA cm**^−2^**)**‡	***V***_**OC**_ **(V)**	**FF**	**PCE**_**max**_ **(PCE**_**ave**_**) (%)**§
PC_61_BM	1:1	195	9.6	10.0	1.0	0.59	5.6 (5.4±0.22)
	1:1.5	185	12.5	12.3	0.99	0.66	8.1 (7.9±0.20)
	1:2	190	14.0	14.1	0.98	0.64	8.7 (8.4±0.25)
	1:3	210	14.6	14.7	0.97	0.62	8.8 (8.5±0.22)
PC_71_BM	1:2	225	14.5	14.5	0.96	0.64	8.9 (8.5±0.20)

EQE, external quantum efficiency; PCE, power conversion efficiency.

^*^Thickness of the active layer.

^†^Short-circuit current density observed from the *J–V* measurement.

^‡^Short-circuit current density calculated from the EQE spectra.

^§^PCE_max_: maximum power conversion efficiency, PCE_ave_: average power conversion efficiency with standard deviation from more than 20 devices.
