# Supplementary material for: High-efficiency polymer solar cells with small photon energy loss
Source: Nat Commun. 2015 Dec 2;6:10085. doi: 10.1038/ncomms10085 (PMC4686756; doi:10.1038/ncomms10085)
Supplement: Supplementary Information — Supplementary Figures 1-13, Supplementary Tables 1-2, Supplementary Notes 1-3, Supplementary Methods and Supplementary References. [file ncomms10085-s1.pdf]

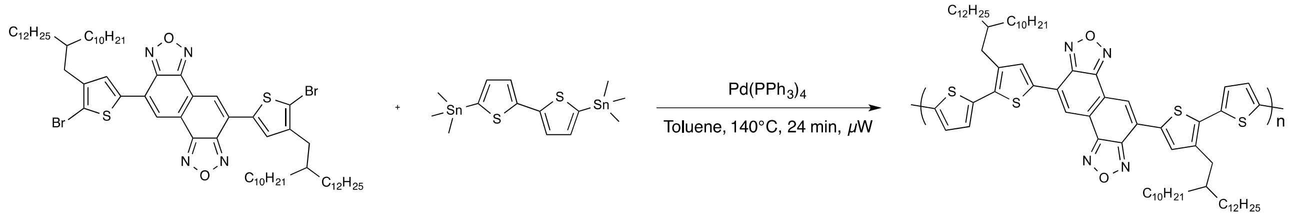

Supplementary Figure 1. Synthetic route to PNOz4T.

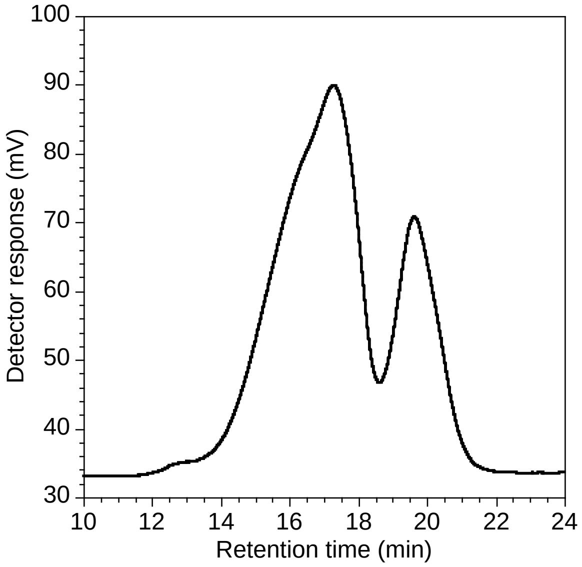

Supplementary Figure 2. GPC chart of PNOz4T. GPC measurement was carried out with a TOSOH HLC-8121GPC/HT instrument at 140 °C using DCB as a solvent. Molecular weights were calibrated with polystyrene standards.

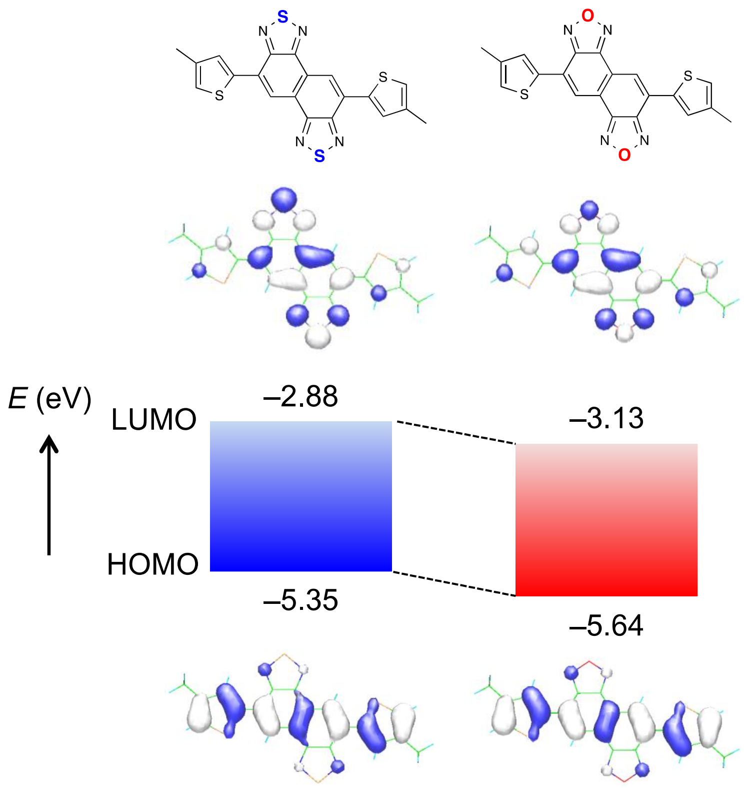

Supplementary Figure 3. HOMOs and LUMOs of the model compound of NTz and NOz calculated with the DFT method at the B3LYP/6-31G(d) level using Gaussian03 program package.

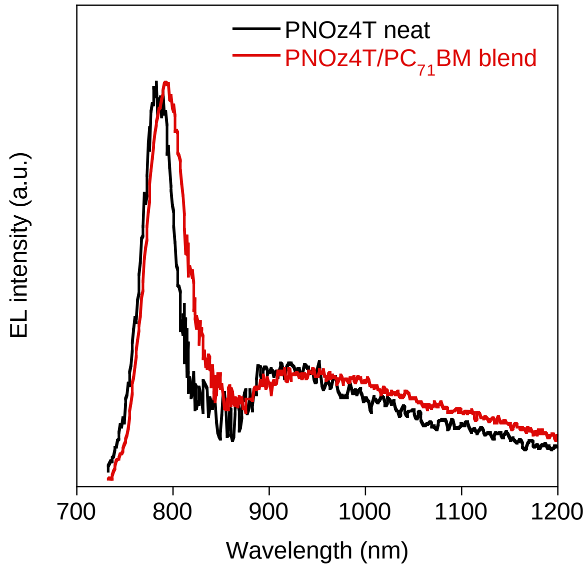

Supplementary Figure 4. EL spectra of the PNOz4T neat film and PNOz4T/$PC_{71}BM$ blend film.

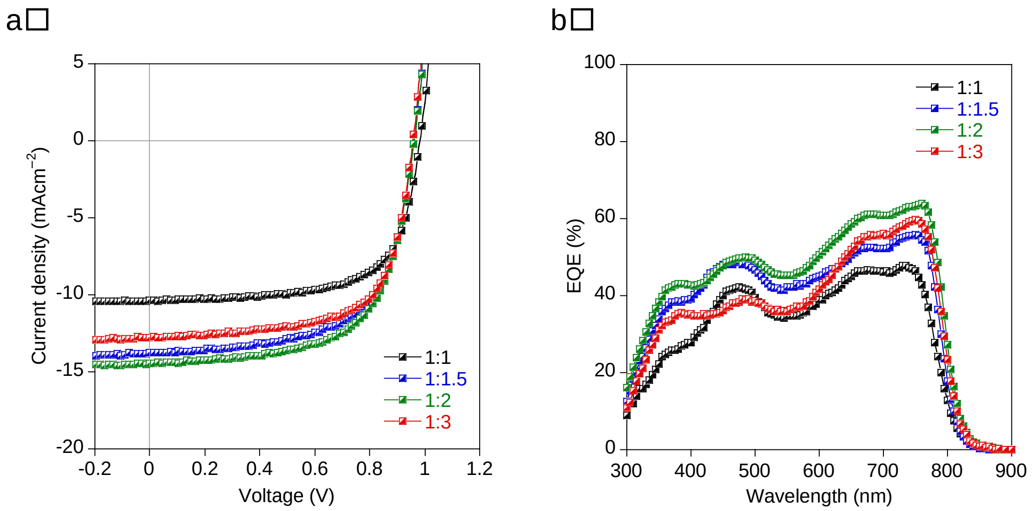

Supplementary Figure 5. *J–V* curves (a) and EQE spectra (b) of PNOz4T/$PC_{71}BM$ cells with the different p/n ratio.

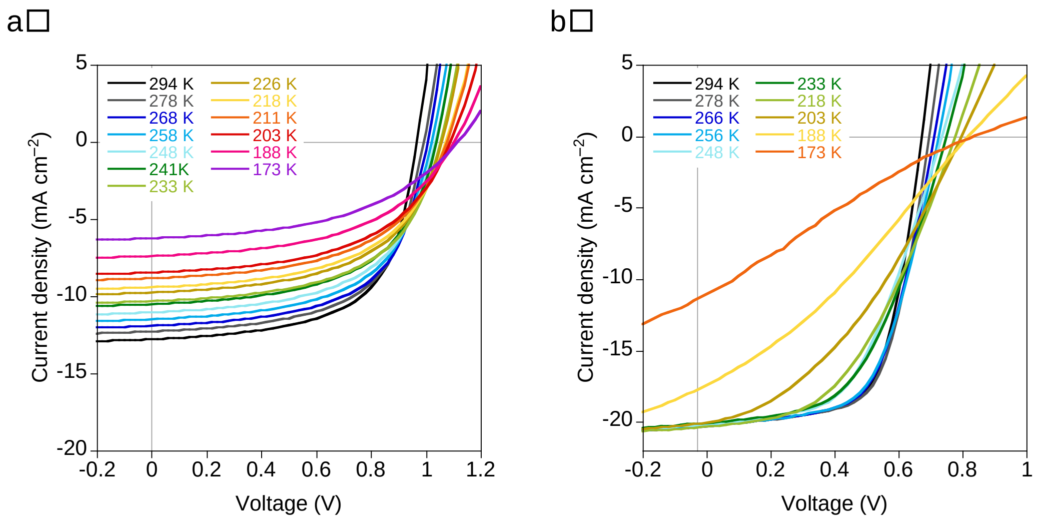

Supplementary Figure 6. Temperature dependent *J–V* curves of the cells with a p/n ratio of 1:2. (a) PNOz4T/$PC_{71}BM$ cell. (b) PNTz4T/$PC_{71}BM$ cell.

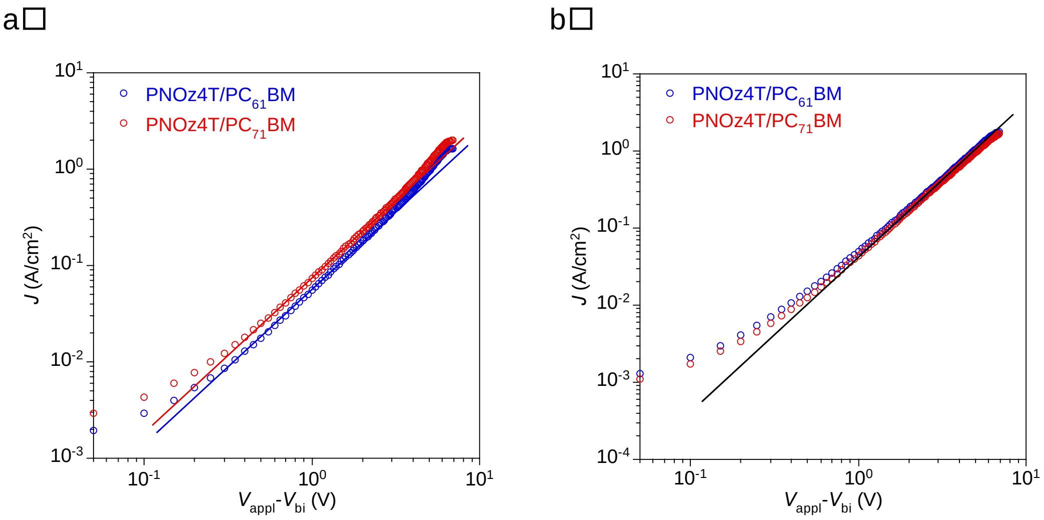

Supplementary Figure 7. *J–V* curves of hole-only devices and electron-only devices of PNOz4T/PCBM films. (a) Hole-only devices: ITO/PEDOT:PSS/PNOz4T:PCBM/MoO_x/Ag. (b) Electron-only devices: ITO/ZnO/PNOz4T:PCBM/LiF/Al. The p/n ratios were 1:3 for $PC_{61}BM$ and 1:2 for $PC_{71}BM$.

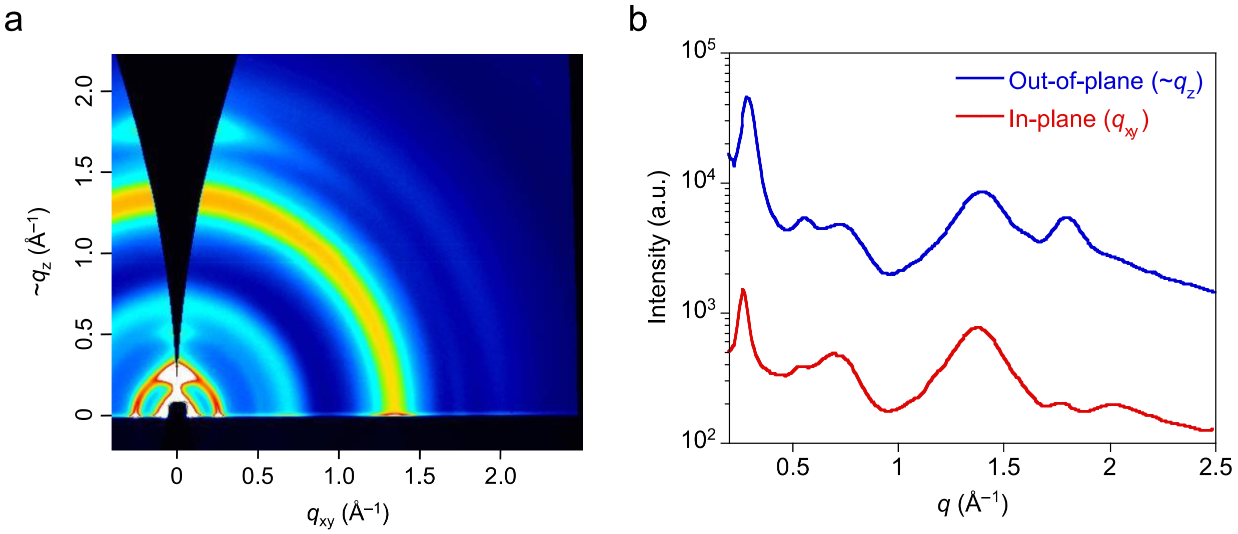

Supplementary Figure 8. 2D-GIXD characterization of PNOz4T/$PC_{61}BM$ (1:3 wt ratio) film spun on ZnO-coated glass/ITO substrate. (a) 2D GIXD image. (b) Cross-sectional profiles of the 2D GIXD image along the q_z (out-of-plane) and q_{xy} (in-plane) axes.

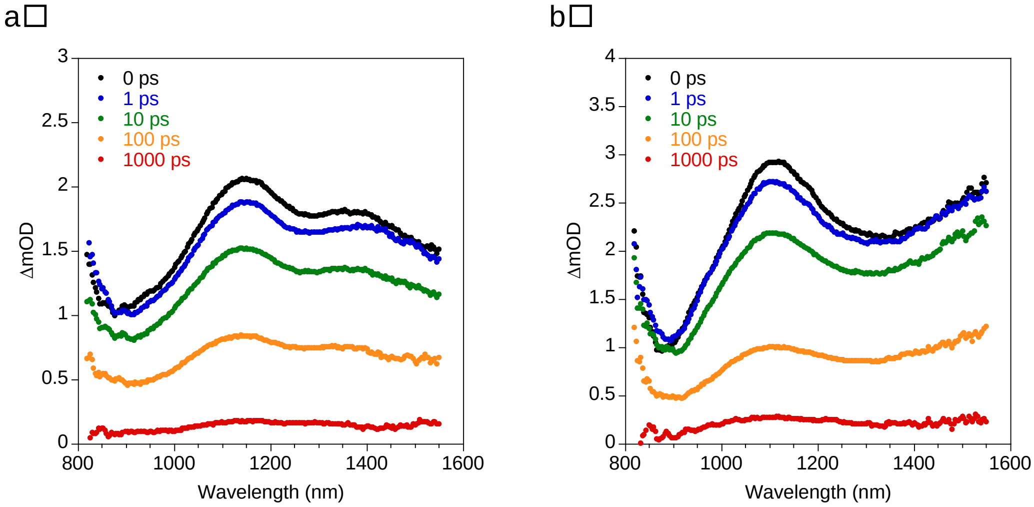

Supplementary Figure 9. Femtosecond transient absorption spectra measured at 0–1000 ps. (a) PNTz4T neat film. (b) PNOz4T neat film. The excitation wavelength was 750 nm for both films.

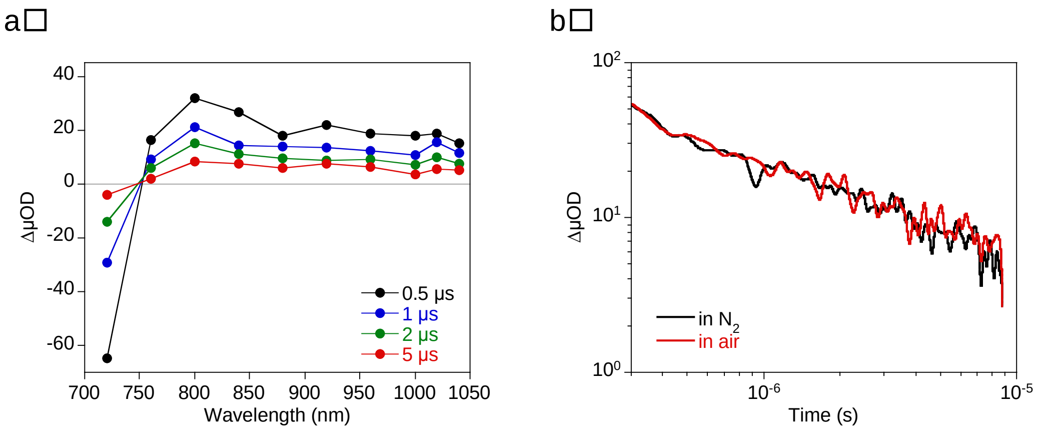

Supplementary Figure 10. Microsecond transient absorption characteristics of the PNOz4T/$PC_{71}BM$ blend film. (a) Microsecond transient absorption spectra measured at 0.5–5 µs. (b) Transient absorption decays measured at 800 nm under an N_2 (black line) and ambient condition (red line).

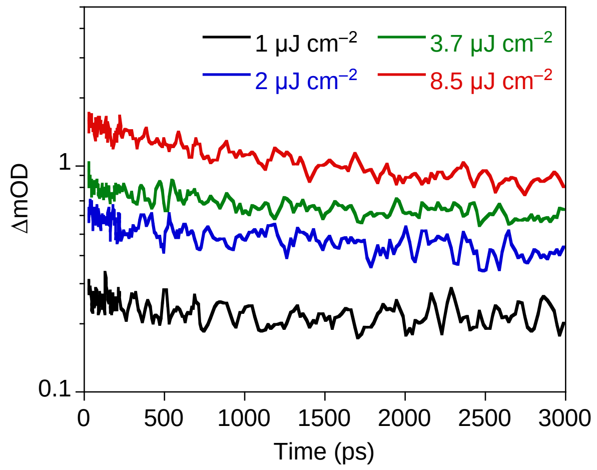

Supplementary Figure 11. Transient absorption decays of the PNTz4T polaron at different excitation intensity.

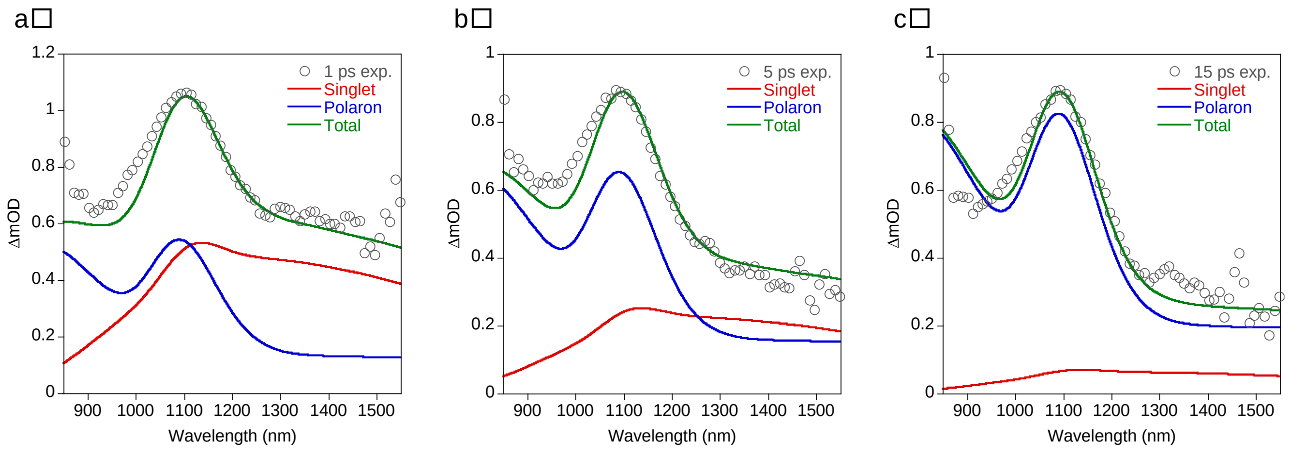

Supplementary Figure 12. Femtosecond transient absorption characteristics of the PNTz4T/PC_{71}BM (1:2 wt ratio) blend film. (a) Spectrum at 1 ps (open circles). (b) Spectrum at 5 ps (open circles). (c) Spectrum at 15 ps (open circles). The green lines represent spectra simulated by the sum of the singlet exciton (red line) and polaron (blue line).

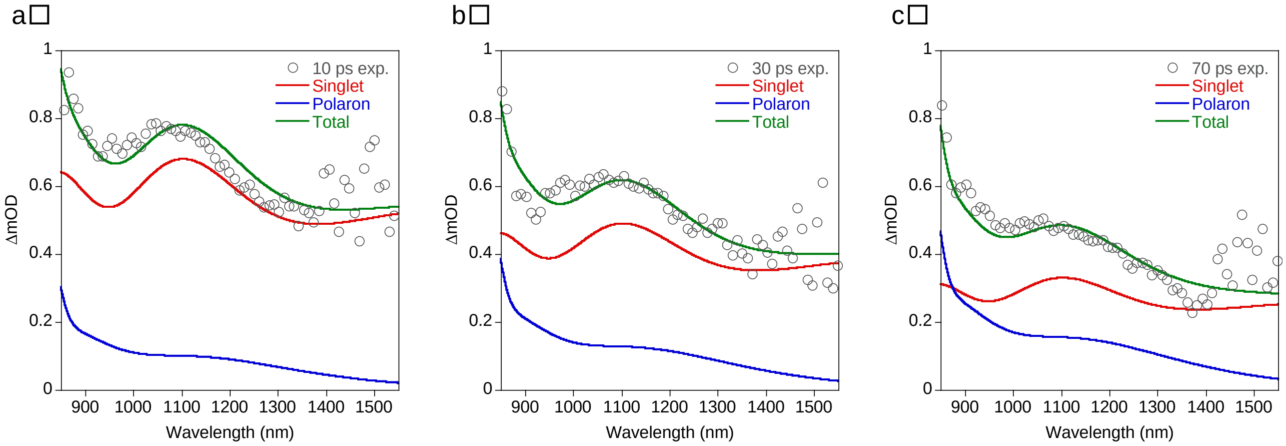

Supplementary Figure 13. Femtosecond transient absorption characteristics of the PNOz4T/$PC_{71}BM$ (1:2 wt ratio) blend film. (a) Spectrum at 10 ps (open circles). (b) Spectrum at 30 ps (open circles). (c) Spectrum at 70 ps (open circles). The green lines represent spectra simulated by the sum of singlet exciton (red line) and polaron (blue line).

Supplementary Table 1. Photovoltaic parameters of PNOz4T /$PC_{71}BM$ cells.

p/n ratio	active layer thickness (nm)	J_{SC} (mA cm^{-2})	V_{OC} (V)	FF	PCE (%)
1:1	145	10.4	0.98	0.67	6.8
1:1.5	190	13.8	0.96	0.64	8.5
1:2	225	14.5	0.96	0.64	8.9
1:3	250	13.0	0.95	0.66	8.2

Supplementary Table 2. E_g, V_{OC}, $E_g - eV_{OC}$, and PCE for various solar cells

System	Compound	E_g (eV)	V_{OC} (V)	$E_g - eV_{OC}$ (eV)	PCE (%)	Supplementary Reference
Inorganic	*c*-Si	1.12	0.71	0.41		1
	GaAs	1.42	1.11	0.31		
	InP	1.28	0.88	0.40		
	CdTe	1.45	0.84	0.61		
	CIGS	1.15	0.72	0.43		
	a-Si	1.73	0.88	0.85		
Perovskite	$CH_3NH_3PbI_{3-x}Cl_x$	1.55	1.1	0.45		2
DSSC	Black Dye	1.3	0.71	0.59		1
	N719	1.6	0.85	0.75		
Organic (Small molecule)	DBP	1.9	0.92	0.98	3.6	3
	CuPc	1.6	0.5	1.1	4.4	4
	DPSQ	1.5	0.91	0.59	4.8	5
	O-BDTdFBT	1.83	0.97	0.86	8.1	6
	BTR	1.82	0.90	0.92	9.3	7
	DR3TSBDT	1.74	0.92	0.82	9.95	8
	DCV5T-Me(3)	1.69	0.95	0.74	6.9	9
	DRCN5T	1.60	0.92	0.68	10.08	10
	$DTS(PTTh_2)_2$	1.52	0.78	0.74	6.7	11
Organic (Polymer)	P3HT/PCBM	1.9	0.61	1.29	4.4	12
	P3HT/indC60	1.9	0.84	1.06	6.5	13
	PCDTBT	1.88	0.88	1.00	6.1	14,15
	PBTI3T	1.81	0.86	0.95	8.66	16
	PM6	1.80	0.98	0.82	9.2	17
	PBDTTPD	1.73	0.97	0.76	8.5	18,19
	PNNT	1.68	0.82	0.86	8.2	20
	PTB7	1.65	0.74	0.91	7.4	21
	PffBT4T-2OD	1.65	0.77	0.88	10.5	22
	TBTIT	1.6	0.72	0.88	9.1	23
	PBDT-TS1	1.51	0.80	0.71	9.48	24
	P3TI	1.50	0.70	0.80	6.3	25

	PTI-1	1.6	0.91	0.69	4.5	26
	PIPCP [e]	1.47	0.86	0.61	6.15	27
	PDPP2Tz2T [d]	1.47	0.92	0.55	5.1	28
	PDPP2TzT [c]	1.44	0.96	0.48	1.1	28
	PSBTBT	1.45	0.68	0.77	5.1	29
	PCPDTBT	1.46	0.62	0.84	5.5	30
	PDPP3T*alt*TPT	1.43	0.75	0.68	8.0	31
	PDPT-DFBT	1.38	0.70	0.68	8.0	32
	PDPP3T	1.33	0.67	0.66	7.1	31
	PBDTDPP [b]	1.31	0.82	0.49	5.16	33
	PDTTDPP [a]	1.22	0.66	0.56	6.05	34
	PDTP-DTDPP	1.13	0.38	0.75	2.71	35
	TTV2	1.1	0.42	0.68	4.99	36
	PNTz4T	1.56	0.71	0.85	10.1	37
	PNOz4T	1.52	0.96	0.56	8.9	This work

[a]–[e] correspond to the plots labeled a–e in Figure 2c and 2d.

Supplementary Note 1.

Synthesis of PNOz4T. 5,10-Bis(5-bromo-4-(2-decyltetradecyl)thiophen-2-yl)naphtho[1,2-*c*:5,6-*c*′]-bis[1,2,5]oxadiazole (120.7 mg, 0.10 mmol), 5,5′-bis(trimethylstannyl)-2,2′-bithiophene (49.2 mg, 0.10 mmol), tetrakis(triphenylphosphine)palladium(0) (2.30 mg, 2 μmol) and dry toluene (4 mL) were added in a 5-mL reaction vessel. The vessel was purged with argon and subsequently sealed. The vessel was put into a microwave reactor and heated to 140 °C for 24 min. After cooling to room temperature, the reaction mixture was poured into a mixture of methanol (200 mL) and concentrated hydrochloric acid (10 mL) and vigorously stirred for 6 h at room temperature. The precipitate was filtered and subjected to sequential Soxhlet extraction with methanol, hexane, chloroform to remove low molecular weight fraction. The residue was extracted with chlorobenzene, concentrated and precipitated in 200 mL of methanol. The precipitate was isolated by filtration, and dried in vacuo to afford PNOz4T (114 mg, 94 %) as metallic purple solids. GPC (DCB, 140 °C): M_n = 57,200, M_w = 417,000, PDI = 7.28. Anal. Calcd for $C_{74}H_{106}N_4O_2S_4$: C, 73.34; H, 8.82; N, 4.62. Found: C, 73.10; H, 8.78; N, 4.38.

Supplementary Note 2.

Definition of E_g. For fair comparison, we carefully double-checked the reference papers for the small molecules and polymers plotted in Figure 2c and 2d (see Supplementary References), if E_g was defined as the onset of the optical absorption. Although some papers do not clearly state the definition, we, in those cases, confirmed by our hands from the absorption spectrum shown in those papers if the absorption onset indeed corresponded to the E_g value. To verify, we here show some data and/or description extracted from the reference papers for the most important polymers with E_{loss} < 0.6 eV, which are labeled as a–e in Figure 2c and 2d.

1. Polymer labeled as "a" (PDTTDPP): E_g of this polymer used in Figure 2c and 2d is 1.22 eV.

Supplementary Ref. 34: Jung, J. W., Liu, F., Russell, T. P. & Jo, W. H. A high mobility conjugated polymer based on dithienothiophene and diketopyrrolopyrrole for organic photovoltaics. *Energy Environ. Sci.* **5**, 6857–6861 (2012).

The authors of this paper stated on the second page of the literature, left column, line 27, as "*the maximum absorption (λ_{max}) at 802 nm and the absorption onset (λ_{onset}) at 1015 nm*." This value (1015 nm) is converted to 1.22 eV, by using the formula, $E = 1240/\lambda$, which is consistent with their statement in the same paragraph "*Thus the electrochemical bandgap ($E_{g,elc}$) was 1.39 eV, which agrees well with the optical bandgap ($E_{g,opt}$) of 1.22 eV*."

2. Polymer labeled as "b" (PBDTDPP): E_g of this polymer used in Figure 2c and 2d is 1.31 eV.

Supplementary Ref. 33: Jung, J. W., Jo, J. W., Liu, F., Russell, T. P. & Jo, W. H. A low band-gap polymer based on unsubstituted benzo[1,2-*b*:4,5-*b*′]dithiophene for high performance organic photovoltaics. *Chem. Commun.* **48**, 6933–6935 (2012).

The figure shown below is extracted from this paper. If we draw the line as shown below in blue, the onset is determined to be ca. 950 nm, which corresponds to E_g of 1.31 eV according to the formula $E = 1240/\lambda$. This value is consistent with the value shown in Table 1 in this paper.

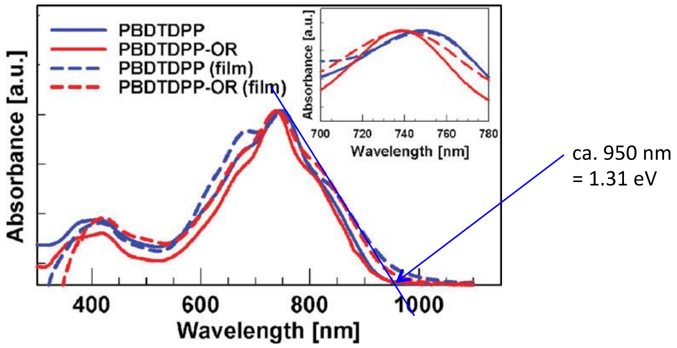

Fig. 1 UV-Vis absorption spectra of PBDTDPP and PBDTDPP-OR. Inset is enlarged spectra in the range of 700–780 nm.

Table 1 Molecular weight, optical, electrochemical and photovoltaic properties of PBDTDPP and PBDTDPP-OR

Polymer	HOMO/eV	LUMO/eV	$E_{g,opt}$/eV	$E_{g,ele}$/eV	V_{OC}/V	J_{SC}/mA cm^{-2}	FF (%)	PCE[a] (%)
PBDTDPP	−5.46	−3.74	1.31	1.72	0.82	10.49	60.0	5.16
PBDTDPP-OR	−5.29	−3.55	1.31	1.74	0.61	9.16	40.2	2.24

[a] PCE values are obtained from optimized devices.

6934 | *Chem. Commun.*, 2012, **48**, 6933–6935 This journal is © The Royal Society of Chemistry 2012

* Both the figure and table shown above are reproduced with permission from Supplementary Ref. 33. Copyright 2012 The Royal Society of Chemistry.

3. Polymer labeled as “c” (PDPP2TzT) and “d” (PDPP2Tz2T): E_gs used in Figure 2c and 2d is 1.44 eV for polymer “c” and 1.47 eV for polymer “d”.

Supplementary Ref. 28: Li, W., Hendriks, K. H., Furlan, A., Wienk, M. M. & Janssen, R. A. J. High quantum efficiencies in polymer solar cells at energy losses below 0.6 eV. *J. Am. Chem. Soc.* **137**, 2231−2234 (2015).

In the first page of this paper, left column, line 5, the authors mention as “*One of the main reasons that the performance of PSCs remains low compared to the best inorganic solution processed thin film solar cells, such as perovskite solar cells, is the significant loss in energy of the open-circuit voltage (V_{OC}) relative to the optical band gap (E_g), determined from the onset of the linear absorption.*”

4. Polymer labeled as “e” (PIPCP): E_g of this polymer used in Figure 2c and 2d is 1.47 eV.

Supplementary Ref. 27: Wang, M. *et al.* High open circuit voltage in regioregular narrow band gap polymer solar cells. *J. Am. Chem. Soc.* **136**, 12576−12579 (2014).

The figure shown below is extracted from the Supporting Information of this paper. As can be seen, the authors determined the absorption onset of this polymer (PIPCP) to be 844 nm. This corresponds to E_g of 1.47 eV according to the formula $E = 1240/\lambda$, which is consistent with the value stated on the first page, right column, line 9 “… *is characterized by an optical band gap (E_g) of ~1.47 eV.*”

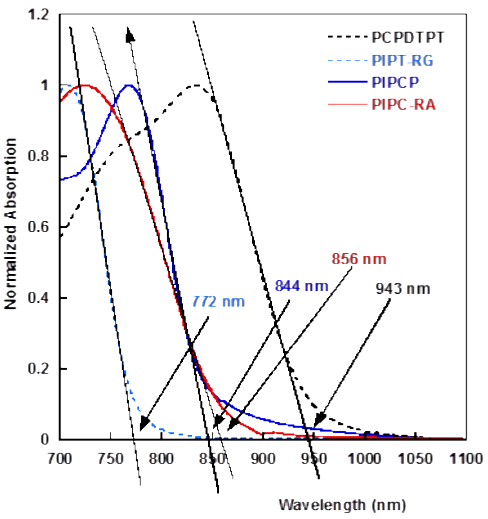

Figure S-5. Film absorption edges of polymers.

Reproduced with permission from Supplementary Ref. 27. Copyright 2014 American Chemical Society.

Supplementary Note 3.

Transient absorption spectra. Supplementary Fig. 9 shows the transient absorption spectra of the polymer neat films measured at 0–1000 ps. Immediately after the photoexcitation at 750 nm, broad absorption bands were observed in the near-IR region. We therefore assign these absorption bands to singlet excitons. Supplementary Fig. 10 shows the microsecond transient absorption spectra and the transient absorption decays measured at 800 nm of the PNOz4T/$PC_{71}BM$ blend film. The broad absorption band observed in the PNOz4T blend film was still observed even on a time scale of microseconds (Supplementary Fig. 10a), and not quenched under an O_2 atmosphere (Supplementary Fig. 10b). Thus, the broad absorption band was not ascribed to triplet excitons but rather to polarons. Supplementary Fig. 11 depicts the transient absorption decays of the PNTz4T polaron at different excitation intensity. The decay dynamics of PNTz4T polaron was independent of the excitation intensity at least <3.7 μJ cm^{-2}, suggesting geminate recombination to the ground state.

To discuss the formation dynamics of polarons, we carefully analyzed the time evolution of transient species on the basis of spectral simulation. The spectral templates of singlet excitons were obtained from the transient absorption spectra of the polymer neat films measured at 0 ps. Those of polarons were obtained from the transient absorption spectra of the blend films measured at 1000 ps. As shown in Supplementary Fig. 12 and 13, the transient absorption spectra observed can be well reproduced by a sum of these absorption templates.

Supplementary Methods

Microsecond transient absorption spectroscopy. Microsecond transient absorption data were collected with a highly sensitive microsecond transient absorption system. A dye laser (Photon Technology International Inc., GL-301) pumped by a nitrogen laser (Photon Technology International Inc., GL-3300) was used as an excitation source, which provides sub-nanoseconds pulses with various fluences from ~μJ to 0.1 mJ cm^{-2} at a repetition rate of 4 Hz. The excitation wavelength was 720 nm. A tungsten lamp (Thermo-Oriel, 66997) with an intensity controller (Thermo-Oriel, 66950) was used as the probe light source. The probe wavelength was selected by two monochromators (Ritsu, MC-10C) and appropriate optical cut-off filters equipped before and after the sample to reduce scattered light and emission. The probe light was detected with a pre-amplified Si photodiode (Costronics Electronics). The detected signal was sent to the main amplification system with an electronic band-pass filter (Costronics Electronics) to improve the signal-to-noise ratio, which was collected by using a digital oscilloscope (Tektronix, TDS2022) synchronized with a trigger signal of the laser pulse from an Si photodiode (Thorlabs, DET10A). The instrument response was of the order of 60 ns. Transient absorption decays were collected over the time range from sub-micro to milliseconds, averaging 4000 laser shots on each delay time scale, yielding a sensitivity of 10^{-6} to 10^{-4} depending on the measuring time domain.

Supplementary References

1. Nayak, P. K., Bisquert, J. & Cahen, D. Assessing possibilities and limits for solar cells. *Adv. Mater.* **23**, 2870–2876 (2011).

2. Snaith, H. Perovskites: the emergence of a new era for low-cost, high-efficiency solar cells. *J. Phys. Chem. Lett.* **4**, 3623–3630 (2013).

3. Fujishima, D. *et al.* Organic thin-film solar cell employing a novel electron-donor material. *Sol. Energy Mater. Sol. Cells* **93**, 1029–1032 (2009).

4. Yang, F., Sun, K. & Forrest, S. R. Efficient solar cells using all-organic nanocrystalline networks. *Adv. Mater.* **19**, 4166–4171 (2007).

5. Wei, G. *et al.* Functionalized squaraine donors for nanocrystalline organic photovoltaics. *ACS Nano* **6**, 972–978 (2012).

6. Yuan, L. *et al.* Oligomeric donor material for high-efficiency organic solar cells: breaking down a polymer. *Adv. Mater.* **27**, 4229–4233 (2015).

7. Sun, K. *et al.* A molecular nematic liquid crystalline material for high-performance organic photovoltaics. *Nat. Commun.* **6**, 6013 (2015).

8. Kan, B. *et al.* Solution-processed organic solar cells based on dialkylthiol-substituted benzodithiophene unit with efficiency near 10%. *J. Am. Chem. Soc.* **136**, 15529−15532 (2014).

9. Fitzner, R. *et al.* Correlation of π-conjugated oligomer structure with film morphology and organic solar cell performance. *J. Am. Chem. Soc.* **134**, 11064−11067 (2012).

10. Kan, B. *et al.* A Series of simple oligomer-like small molecules based on oligothiophenes for solution-processed solar cells with high efficiency. *J. Am. Chem. Soc.* **137**, 3886−3893 (2015).

11. Sun, Y. *et al.* Solution-processed small-molecule solar cells with 6.7% efficiency. *Nat. Mater.* **11**, 44–48 (2011).

12. Li, G. *et al.* High-efficiency solution processable polymer photovoltaic cells by self-organization of polymer blends. *Nat. Mater.* **4**, 864–868 (2005).

13. Zhao, G., He, Y. & Li, Y. 6.5% Efficiency of polymer solar cells based on poly(3-hexylthiophene) and indene-C_{60} bisadduct by device optimization. *Adv. Mater.* **22**, 4355–4358 (2010).

14. Blouin, N. *et al.* Toward a rational design of poly (2,7-carbazole) derivatives for solar cells. *J. Am. Chem. Soc.* **130**, 732–742 (2008).

15. Park, S. H. *et al.* Bulk heterojunction solar cells with internal quantum efficiency approaching 100%. *Nat. Photon.* **3**, 297–303 (2009).

16. Guo, X. *et al.* Polymer solar cells with enhanced fill factors. *Nat. Photon.* **7**, 825–833 (2013).

17. Zhang, M., Guo, X., Ma, W., Ade, H. & Hou, J. A large-bandgap conjugated polymer for versatile photovoltaic applications with high performance. *Adv. Mater.* **27**, 4655–4660 (2015).

18. Cabanetos, C. *et al*. Linear side chains in benzo[1,2-*b*:4,5-*b′*]dithiophene–thieno[3,4-*c*]pyrrole-4,6-dione polymers direct self-assembly and solar cell performance. *J. Am. Chem. Soc.* **135**, 4656–4659 (2013).

19. Piliego, C. *et al.* Synthetic control of structural order in *N*-alkylthieno[3,4-*c*]pyrrole-4,6 -dione-based polymers for efficient solar cells. *J. Am. Chem. Soc.* **132**, 7595–7597 (2010).

20. Osaka, I., Kakara, T., Takemura, N., Koganezawa, T. & Takimiya, K. Naphthodithiophene–naphthobisthiadiazole copolymers for solar cells: alkylation drives the polymer backbone flat and promotes efficiency. *J. Am. Chem. Soc.* **135**, 8834–8837 (2013).

21. Liang, Y. *et al.* For the bright future–bulk heterojunction polymer solar cells withpower conversion efficiency of 7.4%. *Adv. Mater.* **22**, E135–E138 (2010).

22. Liu, Y. *et al.* Aggregation and morphology control enables multiple cases of high-efficiency polymer solar cells. *Nat. Commun.* **5**, 5293 (2014).

23. Yue, W. *et al.* A Thieno[3,2-*b*][1]benzothiophene isoindigo building block for additive- and annealing-free high-performance polymer solar cells. *Adv. Mater.* **27**, 4702–4707 (2015).

24. Ye, L., Zhang, S., Zhao, W., Yao, H. & Hou, J. Highly efficient 2D-conjugated benzodithiophene-based photovoltaic polymer with linear slkylthio side chain. *Chem. Mater.* **26**, 3603−3605 (2014).

25. Wang, E. *et al.* An easily accessible isoindigo-based polymer for high-performance polymer solar cells. *J. Am. Chem. Soc.* **2011**, *133*, 14244–14247.

26. Ma, Z., Wang, E., Vandewal, K., Andersson, M. R. & Zhang, F. Enhanced performance of organic solar cells based on an isoindigo-based copolymer by balancing absorption and miscibility of electron acceptor. *Appl. Phys. Lett.* **99**, 143302 (2011).

27. Wang, M. *et al.* High open circuit voltage in regioregular narrow band gap polymer solar cells. *J. Am. Chem. Soc.* **136**, 12576−12579 (2014).

28. Li, W., Hendriks, K. H., Furlan, A., Wienk, M. M. & Janssen, R. A. J. High quantum efficiencies in polymer solar cells at energy losses below 0.6 eV. *J. Am. Chem. Soc.* **137**, 2231−2234 (2015).

29. Hou, J., Chen, H.-Y., Zhang, S., Li, G. & Yang, Y. Synthesis, characterization, and photovoltaic properties of a low band gap polymer based on silole-containing polythiophenes and 2,1,3-benzothiadiazole. *J. Am. Chem. Soc.* **130**, 16144–16145 (2008).

30. Peet, J. *et al.* Efficiency enhancement in low-bandgap polymer solar cells by processing with alkane dithiols. *Nat. Mater.* **6**, 497–500 (2007).

31. Hendriks, K. H., Heintges, G. H. L., Gevaerts, V. S., Wienk, M. M. & Janssen, R. A. J. High-molecular-weight regular alternating diketopyrrolopyrrole-based terpolymers for efficient organic solar cells. *Angew. Chem. Int. Ed.* **52**, 8341–8344 (2013).

32. Dou, L. *et al.* Synthesis of 5H-dithieno[3,2-*b*:2′,3′-*d*]pyran as an electron-rich building block for donor–acceptor type low-bandgap polymers. *Macromolecules* **46**, 3384–3390 (2013).

33. Jung, J. W., Jo, J. W., Liu, F., Russell, T. P. & Jo, W. H. A low band-gap polymer based on unsubstituted benzo[1,2-*b*:4,5-*b*′]dithiophene for high performance organic photovoltaics. *Chem. Commun.* **48**, 6933–6935 (2012).

34. Jung, J. W., Liu, F., Russell, T. P. & Jo, W. H. A high mobility conjugated polymer based on dithienothiophene and diketopyrrolopyrrole for organic photovoltaics. *Energy Environ. Sci.* **5**, 6857–6861 (2012).

35. Zhou, E. *et al.* Diketopyrrolopyrrole-based semiconducting polymer for photovoltaic device with photocurrent response wavelengths up to 1.1 μm. *Macromolecules* **43**, 821–826 (2010).

36. Zhou, E., Cong, J., Hashimoto, K. & Tajima, K. Introduction of a conjugated side chain as an effective approach to improving donor–acceptor photovoltaic polymers. *Energy Environ. Sci.* **5**, 9756–9759 (2012).

37. Vohra, V. *et al.* Efficient inverted polymer solar cells employing favourable molecular orientation. *Nat. Photon.* **9**, 403–408 (2015).
